# First Report of *Colletotrichum kahawae* Causing Anthracnose on Buckwheat (*Fagopyrum tataricum*) in China and Biological Characterization of the Pathogen

**DOI:** 10.3390/jof11090633

**Published:** 2025-08-29

**Authors:** Xin Liu, Guang Wang, Daowang Sun, Jing Tan, Jiaxing Xie, Binxin Zhai, Chunyan Huang, Wenjie Lu, Lihua Wang

**Affiliations:** 1 Biotechnology and Germplasm Resources Institute, Yunnan Academy of Agricultural Sciences, Kunming 650205, China; 17790631569@163.com (X.L.); wang_guang_17@126.com (G.W.); dwsun03@sina.com (D.S.); 18053241733@163.com (J.X.); xiaozhai943@163.com (B.Z.); hcy456678@163.com (C.H.); luwenjie1976@163.com (W.L.); 2 School of Agriculture, Yunnan University, Kunming 650504, China; tanjing@ynu.edu.cn; 3 Yunnan Provincial Key Lab of Agricultural Biotechnology, Kunming 650504, China; 4 Key Lab of Southwestern Crop Gene Resources and Germplasm Innovation, Ministry of Agriculture, Kunming 650504, China

**Keywords:** *Fagopyrum tataricum*, anthracnose, *Colletotrichum kahawae*, biological characterization

## Abstract

Buckwheat (*Fagopyrum tataricum*) is native to Yunnan, China, and as a miscellaneous grain crop with high nutritional value, it has received increased attention from farmers and enterprises in recent years. In June 2024, we observed severe anthracnose in the buckwheat cultivation area in Malu Township and Jiache Township, Huize County, Qujing City, Yunnan Province, China. In this study, six isolates (SM01–SM06) of anthracnose with similar morphology were obtained using the tissue isolation method, which was due to the fact that this disease is highly pathogenic to buckwheat. The strain SM02 was selected as a representative isolate for biological characterization and molecular phylogenetic analysis, and a phylogenetic tree was constructed based on the *ACT*, *CHS*, and *ITS* genes to determine its taxonomic status. The selected SM02 isolate was further identified as *Colletotrichum kahawae*. Biological characterization showed that the representative strain SM02 exhibited optimal growth for in vitro cultivation under a photoperiod, temperature, pH, carbon source, and nitrogen source of 12L:12D, 25 °C, pH 7.0, glucose, and beef extract, respectively. Host range testing demonstrated that *C. kahawae* might infect important field crops, including maize, wheat, oats, and potatoes. In conclusion, *C. kahawae* causes buckwheat anthracnose in China, which might hinder the production of buckwheat. This study provides insight into anthracnose disease in buckwheat and provides a basis for further investigations to assess and implement effective disease management strategies.

## 1. Introduction

Buckwheat (*Fagopyrum tataricum*) is an annual plant originating from the southwestern part of China that belongs to the Polygonaceae family [1]. Its good qualities include a short growth cycle, tolerance to poor soil, and strong resistance to abiotic stresses [2]. Notably, buckwheat is also considered a healthy cereal food rich in vitamins, minerals, flavonoids, rutin, quercetin, and epicatechin, and it has been extensively planted in China [2,3]. However, the large-scale cultivation of buckwheat has contributed significantly to the occurrence of plant diseases, such as anthracnose [4,5], leaf spot [6], and powdery mildews [7]. Among these diseases, anthracnose is considered to be one of the most serious causes of economic losses to the buckwheat industry [4,5]. Therefore, the prevention and control of buckwheat anthracnose are vital.

Anthracnose is a common plant disease caused by numerous *Colletotrichum* fungal species [8]. Currently, there are approximately 275 commonly accepted *Colletotrichum* species [9]. In tropical and subtropical regions, *Colletotrichum* pathogens infect critical subsistence crops like bananas, cassava, and sorghum, causing significant losses for farmers in developing countries [8,10]. Several *Colletotrichum* species affect high-value crops in temperate and mediterranean regions, such as strawberry [11,12], apple citrus, and olive [13,14]. Species within the genus *Colletotrichum* are also notable for comprising major pathogens responsible for post-harvest decay in fruits and vegetables [15,16]. Only *Colletotrichum liriopes* and *Colletotrichum xishanense* have been reported in buckwheat [4,5], suggesting that the isolation and identification of *Colletotrichum* pathogens on this crop remain insufficient. In addition, some *Colletotrichum* fungi are endophytes of buckwheat, and are correlated with the plant’s flavonoids and phenotypic traits [17,18]. Misidentification of *Colletotrichum* species complexes is a frequent mistake that happens due to few distinctive morphological characters, overlapping morphological characteristics, high genetic diversity, and frequent cryptic speciation [18,19]. The marker set (*ITS*, *ACT*, and *CHS*) was selected based on their proven discriminatory power in previous phylogenetic studies [18,20]. This combination ensures robust species delineation while addressing the limitations of single-gene barcoding in this taxonomically complex genus.

The genus *Colletotrichum* is divided into 16 species complexes and 15 singletons. The *Colletotrichum gloeosporides* species complex (CGSC) is the most species-rich and has the broadest host range [9]. The 56 accepted species within the CGSC can be categorized into three major phylogenetic clades: Musae, Kahawae, and Theobromicola [9,21,22]. Although it is clustered within the CGSC [23], the species *Colletotrichum kahawae* is recognized as a separate taxon because it can infect green coffee leaves and berries at all stages of development [24], as well as other plants, such as *Camellia oleifera* [25], *Hypericum chinensis* [26], and *Cunninghamia lanceolata* [27]. *C. liriopes*- and *C. xishanense*-infected buckwheat have been reported [4,5], but it is unknown whether *C. kahawae* infects buckwheat. Notably, *C. kahawae* is ranked among the ten most significant plant-pathogenic fungi globally. Its high threat level is evidenced by its quarantine status in Asia and Latin America, and its designation as a prohibited biological weapon in Australia [28,29,30]. This disease occurs primarily in Africa, where it severely constrains sustainable Arabica coffee production, causing up to 80% yield losses without fungicide application [29]. Therefore, it is important to prevent and control anthracnose caused by *C. kahawae* for buckwheat production.

Establishing pathogens’ biological characteristics is a fundamental and crucial step in studying disease prevention and control. However, the current research on the biological characteristics of *C. kahawae* is insufficient. For example, Chen et al. reported that *C. kahawae*’s appressorium turgor pressure plays a role in coffee cuticle penetration [31] and that the compounds epicatechin and catechin inhibit the melanization process in *C. kahawae* appressoria [31]. Therefore, investigating the biological characteristics of *C. kahawae* is crucial.

The purposes of this study were to isolate and identify the buckwheat anthracnose pathogen and to determine its biological characteristics. This study provides insight into anthracnose in buckwheat and provides support for the establishment of effective disease management strategies.

## 2. Materials and Methods

### 2.1. Isolation and Purification of Pathogens

In 2024, anthracnose was observed on the leaves of buckwheat *F. tataricum* at multiple buckwheat cultivation sites, including Malu Township (25°59′ N, 103°24′ E, 2476.9 m altitude) and Jiache Township (25°59′ N, 103°24′ E, 2473.3 m altitude), Huize County, Qujing City, Yunnan Province, China. Five fields (total area: 2236 m^2^) of Malu Township and six fields (total area: 2000 m^2^) of Jiache Township were used to calculate the incidence rate. The samples were transported to the laboratory in sealed zip-lock bags for fungal isolation. Pathogenic fungi were isolated and purified from the interface between healthy and diseased leaf tissues using the tissue isolation method [32]. Leaf samples with diseased spots were washed with sterile water and blotted dry, and the diseased parts (3–5 × 3–5 mm) were excised at the junction of the disease. These samples were then surface-sterilized by sequential immersion in 75% ethanol and 2% sodium hypochlorite, each for 1 min, followed by three rinses with sterile water and drying on sterile filter paper. Subsequently, the tissue fragments were transferred to potato dextrose agar (PDA: potato extract 200 g/L, 2% glucose plus 1.5% agar) and incubated at 25 °C in darkness until mycelium emerged [33]. The mycelium around the sterilized leaf tissue was selected for further purification. Purified strains with more than three consecutive subcultures were obtained. Finally, six isolates (SM01–SM06) were obtained, and the strain SM02 was selected, based on its typical pathological features in the field and good growth characteristics on PDA, as a representative isolate for pathogenicity tests, biological characterization, and molecular phylogenetic analysis.

### 2.2. Pathogenicity Tests of Isolate

The isolated strains were cultured on PDA medium and incubated at 25 °C in the dark for 10 days. A sterile hole puncher was used to take mycelial plugs (Φ = 5 mm) from the colony. The mycelium block was inoculated into the wounds of buckwheat leaves with a sterile needle puncture. The inoculated leaves were maintained at 25 °C and monitored for symptom development. After lesions appeared, pathogens were re-isolated from the diseased tissue and identified to fulfill Koch’s postulates. Plain PDA blocks were used as a control (CK), and they were inoculated onto buckwheat leaf wounds. In pathogenicity testing, the buckwheat leaves were healthy and all had the same growth status, and the entire experiment was performed in triplicate.

For the host range test, the leaves of maize (*Zea mays*; jointing stage), wheat (*Triticum aestivum*; heading stage), potato (*Solanum tuberosum*; current bud stage), and oats (*Avena sativa*; heading stage) were obtained from the Songming Experimental Demonstration Base of Yunnan Academy of Agricultural Sciences. Healthy samples were surface-sterilized by rinsing with sterile water, 30 sec sterilization in 75% ethanol, and 2 min immersion in 2.0% NaClO. They were then rinsed more than three times with sterile water. The samples were prepared as follows: (1) A sterile toothpick was used to puncture the wound on the plant. Fungal blocks (Φ = 5 mm) were plucked from the margins of 7-day-old colonies and placed onto the wounded sites of the detached leaves. Plain PDA blocks were inoculated onto the wounds of buckwheat leaves as a control (CK). (2) Healthy and non-wounded leaves received a 2 × 10^6^ conidia suspension treatment. Conidial suspension in 0.02% Tween 80 water was prepared from 7-day-old cultures. Healthy and non-wounded leaves received 0.02% Tween 80 water treatment as a control (CK). We maintained the inoculated leaves in a humid chamber (Petri dish with wet filter paper) at 25 °C for 5 days. We monitored all samples daily, recording and photographing any symptoms. Each treatment was replicated three times.

### 2.3. Morphological Identification of Fungal Isolates

The strain SM02 was inoculated onto PDA medium and incubated at 25 °C in the dark. Colony growth and morphology were observed and photographed daily. Meanwhile, the colony characteristics, mycelial structure, and spores were examined using a light microscope and preserved and recorded through photography. Spore size was measured using Image J software (version 1.54; National Institutes of Health). Spores were then inoculated onto PDA to observe germination after 24 h. The identification of the anthracnose pathogen was confirmed through a combination of cultural characteristics and morphological features.

### 2.4. Molecular Identification of Fungal Isolates

The strain SM02 was inoculated onto PDA medium at 25 °C in the dark for 10 days. Fungal Genomic DNA Rapid Extraction Kit (Qingke Biotechnology, Kunming, China) was used to extract DNA, and polymerase chain reaction (PCR) was performed to amplify *ITS*, *ACT*, and *CHS* using universal barcoding primer pairs (Table S1). PCR was carried out as described in the 2×ApexHF FS PCR Master Mix* (Accurate Biology, Changsha, China) following the manufacturer’s protocol. The integrity of the PCR products was verified by 1.0% agarose gel electrophoresis prior to submission to Qingke Biotechnology Co., Ltd. (Kunming, China) for sequencing. We submitted these sequences to NCBI and obtained the accession numbers. *ITS*, *ACT*, and *CHS* sequences for the same genus and species, for different species of the same genus, and for different species of different genera were downloaded from GenBank, and a phylogenetic tree was constructed together with the sequencing results of SM02. We list all strains used in this study and their corresponding GenBank accession numbers in Table S2. These sequences were aligned and adjusted using the Sequence Processing Online Toolkit (SMS) BioSoft Translation (http://www.bio-soft.net/sms/, accessed on 5 March 2024). We performed the phylogenetic analysis of the SM02 strain based on the combined sequence datasets of *ACT* + *CHS* + *ITS* using the Maximum Likelihood method with a best-fit substitution model, and the phylogenetic tree was generated using MEGA 7.0 software with 1000 bootstrap replicates.

### 2.5. Biological Characterization Studies

After inoculating the mycelium onto PDA medium, the plates were cultured at 25 °C in the dark for 7 days [34]. Mycelial blocks (Φ = 5 mm) were obtained by excising them from the colony edges using a sterile perforator. The mycelial side of the mycelium blocks was placed in the center of the medium and subjected to treatments with various photoperiods, pH values, temperatures, and carbon and nitrogen sources. Each treatment was replicated three times, and colony diameter was measured using the criss-cross method. The various testing methods were as follows.

Effect of Different Photoperiods on Fungal Growth: The PDA medium plates were cultured at 25 °C with different photoperiods (24L:0D, 12L:12D, and 0L:24D) in a culture chamber for 7 days. The growth of the colony was observed, and the colony diameters were measured and photographed.

Effect of Different Temperatures on Fungal Growth: PDA plates were incubated in the dark for 7 days across a temperature range of 5 to 35 °C, including 5 °C, 10 °C, 15 °C, 20 °C, 25 °C, 28 °C, 30 °C, 32 °C, and 35 °C.

Effect of Different pH Values on Fungal Growth: The pH of the PDA medium was adjusted to values ranging from 4 to 11 (4, 5, 6, 7, 8, 9, 10, and 11) using 0.1 mol/L HCl and 0.1 mol/L NaOH. These medium plates were incubated at 25 °C in the dark in a culture chamber. The colony diameter was measured and photographed after 7 days.

Effect of Different Carbon and Nitrogen Sources on Fungal Growth: Czapek’s medium (CZA: 3% sucrose, 0.3% NaNO_3_, 0.1%K_2_HPO_4_, 0.05% KCl, 0.05% MgSO_4_, and 0.001% FeSO_4_ plus 1.5% agar) was used as the basal medium of all formulations, and each formulation contained an equal amount of either glucose, xylose, maltose, lactose, sucrose, or soluble starch as the sole carbon source instead of sucrose, and an equal amount of NH_4_Cl, tryptone, beef paste, urea, yeast, or KNO_3_ as the sole nitrogen source instead of NaNO_3_ [35]. The mycelium blocks were placed in the center of the medium plates containing each carbon and nitrogen source formulation and incubated at 25 °C in dark for 7 days. Colony growth was monitored and recorded periodically.

### 2.6. Statistical Analysis

All data are expressed as mean ± standard deviation (SD). Normality and homoscedasticity of the data were assessed using the Shapiro–Wilk test and Levene’s test, respectively. Statistical comparisons among groups were performed by one-way analysis of variance (ANOVA) followed by Tukey’s post hoc test. All analyses were conducted using IBM SPSS Statistics (version 27.0.1), and graphs were plotted using GraphPad Prism version (version 8.0; San Diego, CA, USA).

## 3. Results

### 3.1. Natural Symptoms

**Figure 1:**
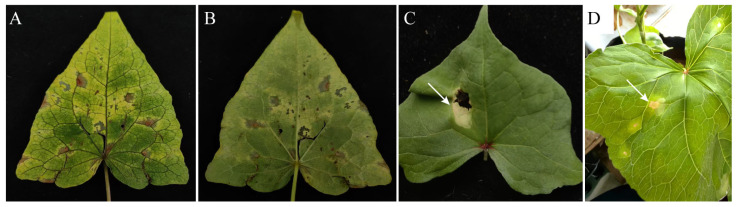
Symptoms of anthracnose development on buckwheat leaves. Symptoms on the upper (**A**) and lower (**B**) sides of diseased leaves in nature. (**C**,**D**) Symptoms of leaves after indoor inoculation; the white arrows point to the symptoms.

In 2024, symptoms of anthracnose were observed in common buckwheat fields across Malu Township and Jiache Township. Among monitored fields, five sites in Malu Township (total area: 2236 m^2^) showed 27.23–32.49% symptomatic plants, while six sites in Jiache Township (total area: 2000 m^2^) exhibited 22.58–25.21% symptomatic plants. Characteristic anthracnose lesions were present on >50% of leaves from affected plants. Buckwheat anthracnose leaf symptoms: Extensive brown spots appear on the leaves, with yellow coloration around the spots. Spots have an irregular or nearly round shape, with whorls, and the central part of the lesion is white to light yellow-brown (Figure 1A,B).

### 3.2. Pathogenicity Tests

Isolated pathogens were inoculated onto the leaves of live, healthy buckwheat plants. Seven days post-inoculation, brown spots appeared on the leaves and yellow coloration around the spots (Figure 1C,D), consistent with symptoms observed in the field. The controls remained asymptomatic. The same fungus was consistently re-isolated from the symptomatic tissues, thereby satisfying Koch’s postulates.

### 3.3. Morphology of Fungal Isolates

**Figure 2:**
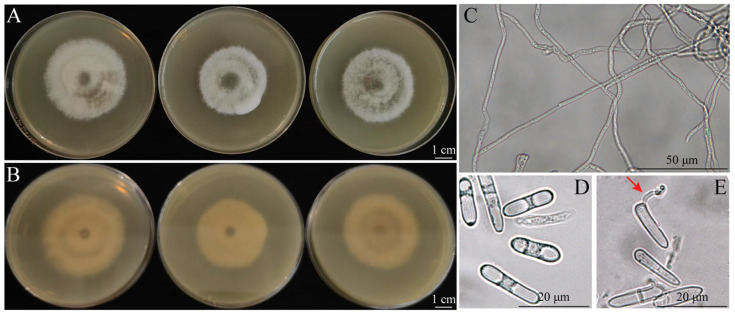
Morphological characteristics of *Colletotrichum* SM02 isolates on PDA. (**A**) Front side of the colony on PDA medium, bar = 1 cm. (**B**) Reverse side of the colony on PDA medium, bar = 1 cm. (**C**) Hyphae morphology, bar = 50 μm. (**D**) Conidia morphology, bar = 20 μm. (**E**) Conidia germination, bar = 20 μm. The red arrow points to the germ tube.

The SM02 isolate exhibited a gray-white colony morphology on PDA medium initially, which later transitioned to an olive-green coloration, with abundant aerial mycelia on the upper side, and spread to the edge (Figure 2A). On the reverse side, the colony exhibited a brown center and produced conspicuous concentric rings (Figure 2B). Hyphae were hyaline to brown and septate (Figure 2C). Conidia were cylindrical, hyaline, smooth-walled, ad aseptate, with rounded ends (Figure 2D) and with a size of 9.86 to 21.59 × 2.31 to 4.77 µm (mean = 16.56 × 3.93 µm, *n* = 52). Spores were germinated 24 h after inoculation on PDA (Figure 2E).

### 3.4. Phylogenetic Analysis

**Figure 3:**
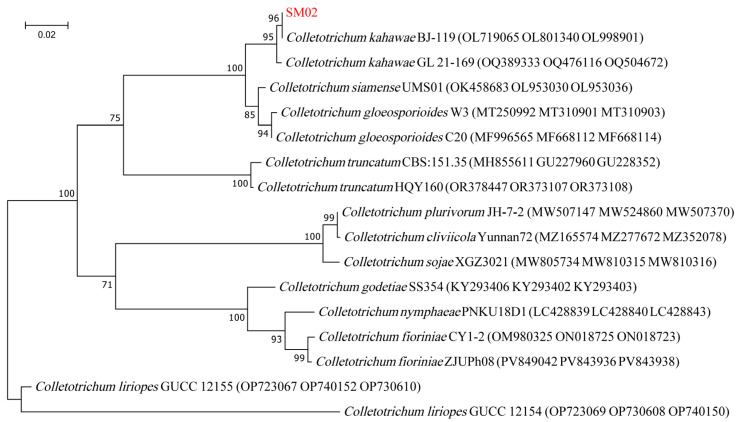
Phylogenetic analysis of *C. kahawae* based on the combined sequence datasets of *ITS* + *CHS* + *ACT* obtained using the Maximum Likelihood method with a Tamura–Nei substitution model. The phylogenetic tree was generated using MEGA 7.0 software with 1000 bootstrap replicates. The red font denotes the SM02 strain determined in this study.

The *ITS*, *CHS*, and *ACT* genes had lengths of 582, 299, and 271 bp and were submitted to NCBI under accession numbers PV545913, PV553736, and PV553737, respectively. NCBI analysis revealed that the isolate shared over 99% sequence homology with *Colletotrichum* sp. Through near-origin comparison, *Colletotrichum* sp. could be used to construct a phylogenetic tree, which based on the combined sequence datasets of *ITS* + *CHS* + *ACT* obtained using the Maximum Likelihood method, showed that the strain SM02 was clustered in the same branch as *C. kahawae* (Figure 3). In addition, we also constructed separate phylogenetic trees based on *ITS* (Figure S1), *ACT* (Figure S2), and *CHS* (Figure S3) using the Maximum Likelihood method. The strain SM02 was also clustered in the same branch as *C. kahawae*. Based on integrated morphological and molecular characterization, the SM02 strain was identified as *C. kahawae*. *C. liriopes* is also a pathogen of buckwheat [4], and *C. liriopes* and *C. kahawae* form independent branches in the phylogenetic tree (Figure 3 and Figures S1–S3). Blast analysis indicated that the *ITS*, *CHS*, and *ACT* sequences of SM02 exhibited 93.07%, 90.17%, and 80.43% identity to their best match accession of *ITS* (MZ314513), *CHS* (KY995452), and *ACT* (KY995503) in *C. liriopes*, respectively (Table S3). The Maximum Likelihood method showed significantly high node support values compared with *C. liriopes* (Table S3).

### 3.5. Biological Characterizations

#### 3.5.1. Effects of Photoperiod on Growth

**Figure 4:**
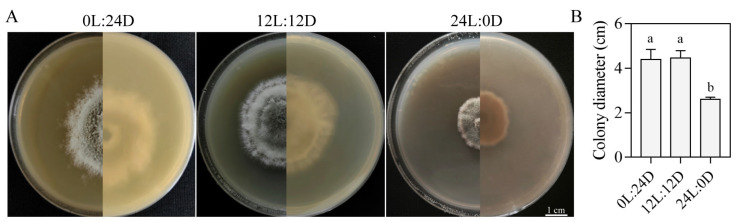
Growth of strain SM02 under different photoperiods. Colony growth images (**A**) and diameters (**B**). Data are presented as mean ± SD. Different letters indicate significant differences (Tukey’s post hoc test, *p* < 0.05). Bar = 1 cm.

After 7 days of incubation, mycelial growth of SM02 under different photoperiods (24L:0D, 12L:12D, and 0L:24D) was measured. Among these treatments, the difference between the 12L:12D and 0L:24D groups was not statistically significant (Figure 4). Colony growth in the 24L:0D group was significantly decreased compared to that in the 12L:12D and 0L:24D groups (Figure 4). The mean colony diameters for the 24L:0D, 12L:12D, and 0L:24D groups were 2.50 cm, 4.70 cm, and 4.40 cm, respectively (Figure 4B). These findings suggest that the optimal photoperiod conditions for *C. kahawae* growth were 12L:12D and 0L:24D.

#### 3.5.2. Growth Response to Temperatures

**Figure 5:**
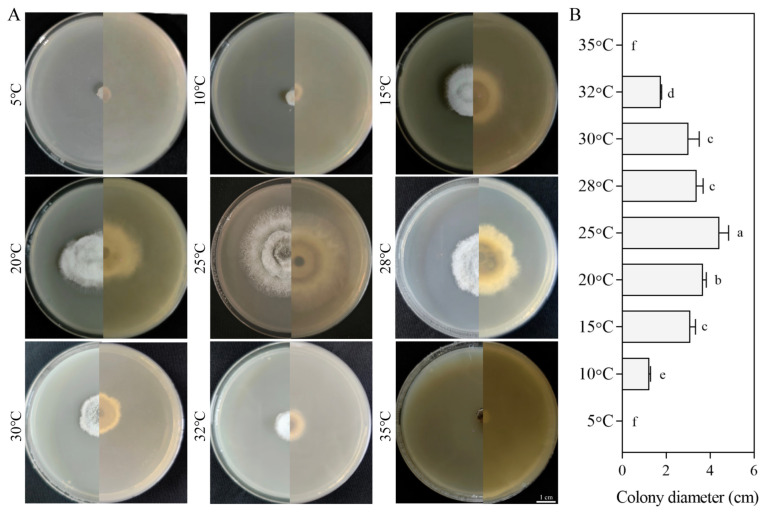
Growth of strain SM02 at different temperatures. Colony growth images (**A**) and diameters (**B**). Data are presented as mean ± SD. Different letters indicate significant differences (Tukey’s post hoc test, *p* < 0.05). Bar = 1 cm.

The SM02 strain exhibited no growth at 5 °C or 35 °C, with a growth range between 10 °C and 32 °C. The growth of the SM02 strain initially increased and then decreased with increasing temperature, peaking at 25 °C, followed by a decline. Mycelial growth decreased significantly beyond 28 °C (Figure 5). At 10 °C and 32 °C, the strain grew extremely slowly with virtually no observable growth, yielding colony diameters of only 1.11 cm and 1.73 cm, respectively (Figure 5B). These results indicate that *C. kahawae* grows within a temperature range of 10 °C to 32 °C, with an optimal temperature of 25 °C.

#### 3.5.3. Effect of pH on Growth

**Figure 6:**
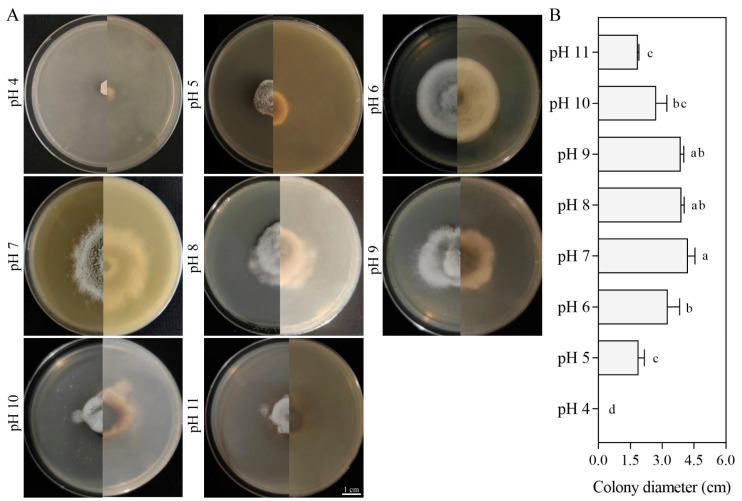
Growth of strain SM02 at different pH values. Colony growth images (**A**) and diameters (**B**). Data are presented as mean ± SD. Different letters indicate significant differences (Tukey’s post hoc test, *p* < 0.05). Bar = 1 cm.

SM02 strain growth did not differ significantly between pH 7 and pH 9, although the strains grew slowly at pH 5, pH 8, pH 10, and pH 11. The growth pattern was relatively stable in this pH range, with optimal growth observed at pH 7, reaching a diameter of 4.55 cm by the seventh day. In contrast, complete growth inhibition occurred at pH 4, and no mycelial development was observed either initially after inoculation by day 7 (Figure 6). Overall, an increase in pH was positively correlated with the growth rate of the colonies, suggesting that *C. kahawae* exhibits greater resistance to alkaline environments.

#### 3.5.4. Growth Characteristics on Different Carbon Sources

**Figure 7:**
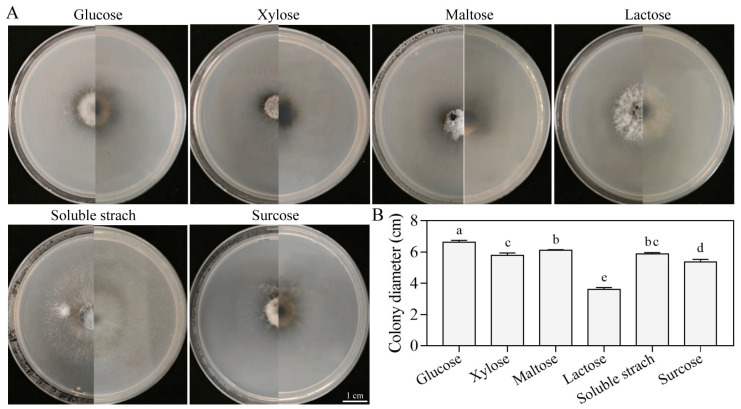
Vegetative growth of strain SM02 under different carbon sources. Colony growth images (**A**) and diameters (**B**). Data are presented as mean ± SD. Different letters indicate significant differences (Tukey’s post hoc test, *p* < 0.05). Bar = 1 cm.

The strain SM02 was cultured on media supplemented with six distinct carbon sources, showing variations in growth rates and colony morphology. Most colonies appeared grayish-white and round, whereas those grown on lactose exhibited a grayish-green color (Figure 7A). Glucose and lactose served as the best and worst carbon sources for mycelial growth, yielding colony diameters of 6.61 cm and 3.62 cm, respectively (Figure 7B). Of the carbon sources tested, glucose yielded the highest mycelial growth rate and largest colony diameter, followed by maltose, soluble starch, xylose, sucrose, and lactose, in descending order. These data indicate that glucose is the optimal carbon source for *C. kahawae* growth.

#### 3.5.5. Growth Characteristics on Different Nitrogen Sources

**Figure 8:**
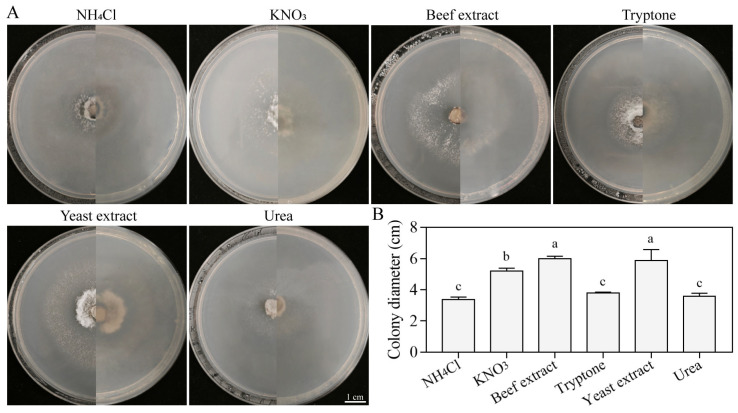
Vegetative growth of strain SM02 under different nitrogen sources. Colony growth images (**A**) and diameters (**B**). Data are presented as mean ± SD. Different letters indicate significant differences at *p* < 0.05, according to Tukey’s post hoc test. Bar = 1 cm.

The growth capacity of the strain SM02 was assessed using media supplemented with various nitrogen source. The results indicated significant variations in both colony morphology and size across the six nitrogen sources (Figure 8A). Beef extract and NH_4_Cl were the optimal and poorest nitrogen sources for mycelial growth, yielding colony diameters of 6.11 cm and 3.25 cm, respectively (Figure 8B). The colony diameters decreased in the following order: beef extract > yeast extract > KNO_3_ > tryptone > urea > NH_4_Cl (Figure 8B). These findings indicate that beef extract is the optimal nitrogen source for *C. kahawae* mycelial growth.

### 3.6. Pathogenicity Test for Four Crop Plants

**Figure 9:**
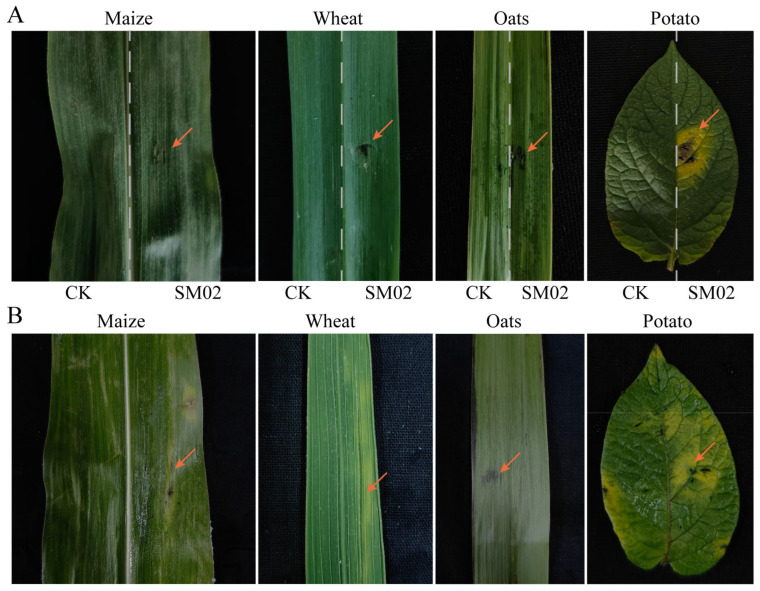
SM02 strain pathogenicity tests on plants in the field. (**A**) Pathogenicity testing of wounded leaves. On the left is the CK leaf and on the right leaf is the treated leaf. (**B**) Pathogenicity testing of non-wounded leaves. The orange arrow points to the locations of the disease.

Since other *Polygonum* spp. are not widely cultivated, to test the host range of the representative strains, we selected field cash crops such as maize, wheat, potato, and oats for host range studies and conducted inoculation experiments on their leaves (Figure 9). Two days after inoculation, black spots appeared on the potato leaves. These infected areas expanded significantly after 4 days, resulting in extensive wilting of the leaves. However, on oat and wheat leaves, black and brown lesions were observed three days after inoculation, with visible darkening near the wound (Figure 9A). During spray treatment, non-wounded leaves developed black spots in oats and yellow spots in wheat (Figure 9B). The symptoms on oat leaves continued to expand and took on an irregular shape after 5 days. No effect was evident on maize leaves after 3 days of inoculation, but at 5 days, their wounds showed black, irregular spots (Figure 9A). Non-wounded corn leaves and wounded corn leaves exhibited the same symptoms (Figure 9B). with similar symptoms appearing on leaves after wound inoculation. Thus, the isolated SM02 strain might be pathogenic to potato, oat, wheat, and maize.

## 4. Discussion

As the cultivation of buckwheat expands, anthracnose is becoming an increasingly serious disease. Anthracnose affects plant growth and appearance, often causing plant death, thereby threatening the development and ecological balance of the buckwheat industry. In our study, *C. kahawae* was isolated from diseased buckwheat leaves and identified by its morphological and molecular features. *C. kahawae* exhibits low genetic variability and is structured into three distinct clonal populations: Angolan, Cameroonian, and East African [36,37], and two clonal lineages within the Angolan population. Moreover, significant differences in isolate aggressiveness were consistently detected, independent of their geographical origin [38,39,40]. Previous studies have reported that *C. kahawae* infects green coffee berries, *C. oleifera* [25], *H. chinensis* [26], and *C. lanceolata* [27], which grow on shrubs or trees. However, buckwheat is an herbaceous plant, suggesting that the host range of *C. kahawae* from different geographic origin also varies. Notably, Yunnan in China is also a major area for coffee cultivation, and *C. kahawae* might pose a threat to the coffee industry in this region. This study provides insight into anthracnose diseases in buckwheat and provides a basis for further investigation to assess and implement effective disease management strategies.

Environmental factors such as temperature, pH, nutrition, and photoperiod are the key factors affecting fungal growth and development. In this study, the optimal photoperiod, temperature, pH, carbon source, and nitrogen source for in vitro cultivation were 12L:12D, 25 °C, pH 7.0, glucose, and beef extract, respectively. The morphological features of the anthracnose pathogen, especially the colony morphology and the creamy white aerial mycelium, are in agreement with previous species descriptions of *Colletotrichum* by Damm et al. [41] and Weir et al. [22]. The optimal temperature for mycelial growth of 25 °C in the present study was similar to that determined by Chen et al. [42] and Bardas et al. [43], and the optimal pH of 7 aligns with results reported by Li et al. [44]. However, the optimum photoperiod was slightly different from that reported by Tao et al. [45]. These results indicate that *C. kahawae* has the same characteristics as most species in the genus *Colletotrichum*.

Its global distribution highlights that the CGSC is present in most of Asia, including Japan and Indonesia; Australia and New Zealand; most of the Americas; and Europe. Notably, the buckwheat cultivation areas are mainly in Asia and Europe [1]. The majority of *Colletotrichum* spp. exhibit growth in temperatures ranging from 10 °C to 35 °C. Sporulation typically occurs around 25 °C for most species in the CGSC [46]. Conidial germination temperatures are much higher in the CGSC, with an optimal temperature range of 25 °C to 31 °C [47]. In this study, *C. kahawae* exhibited growth in temperatures ranging from 10 °C to 35 °C, with an optimal temperature of 25 °C. The buckwheat cultivation areas overlap with the geographical distribution of the CGSC, and the growth and development temperature of *C. kahawae* is consistent with that of the CGSC, suggesting that anthracnose caused by *C. kahawae* might occur in buckwheat cultivation areas through rain splashing and transmission by insects [48]. Previous studies by our research team and other authors have demonstrated that *C. kahawae*, *C. liriopes*, and *C. xishanense* infected buckwheat in the genus *Colletotrichum* [4,5]. *C. kahawae* and *C. xishanense* belong to the CGSC, and *C. liriopes* belongs to the *Colletotrichum spaethianum* species complex (CSSC) [5,9]. The CGSC has been documented to infect 283 plant species across 212 genera, with eudicots comprising the majority (80.6%), while monocots and gymnosperms account for a smaller proportion (16.1% and 2.2%, respectively) [9]. The CSSC is an aggregate containing five species, four of which are associated with petaloid monocot plants [9]. The hosts of CGSC and CSSC are different.

Several previous studies have shown that certain species within the genus *Colletotrichum* can function as weak or conditional pathogens of plants and sources of pathogenic leaf spot pathogens, endophytes, and secondary metabolites [49,50,51]. *C. kahawae* induces a variety of plant diseases, such as root rot in apple [52], anthracnose in African Arabica coffee [53], and anthracnose in *Vietnamese chillies* [54]. In addition, our study found that *C. kahawae* might infect important field crops, including maize, wheat, oats, and potatoes. However, the mechanisms by which anthracnose spp. cause plant diseases remain uncertain. The pathogenicity of *C. kahawae* was studied through comparative genomic analysis with its non-pathogenic sibling species, revealing that its virulence involves a complex biological mechanism, potentially encompassing critical functions such as detoxification and transport, regulation of host and pathogen gene expression, and signaling [55]. In future studies, we will endeavor to unravel the pathogenic mechanism by which these fungal isolates cause anthracnose in buckwheat in China.

## 5. Conclusions

In conclusion, based on morphological observations, molecular characterization, and pathogenicity tests, we determined that *C. kahawae* causes buckwheat anthracnose in Yunnan, China. The optimal photoperiod, temperature, pH, carbon source, and nitrogen source for fungal growth were found to be 12L:12D, 25 °C, pH 7.0, glucose, and beef extract. The optimal biological characteristics of *C. kahawae* provide a basis for monitoring the range of anthracnose occurrence and integrated pest management. However, this study also raises several issues that require clarification in future investigations. Firstly, this study only determined that *C. kahawae* caused anthracnose in buckwheat and its biological characterization. Control strategies and the selection resistant varieties should be investigated in the future. Secondly, we only identified one pathogen of buckwheat anthracnose in Huize County of Yunnan, and there were relatively few isolated strains. Further studies should aim to identify more buckwheat anthracnose pathogens in Yunnan province. Finally, the host range of *C. kahawae* in Yunnan’s economically important plants, including coffee, flowers, vegetables, and Chinese medicinal herbs, should also be investigated in the future.

## Data Availability

The original contributions presented in this study are included in the article. Further inquiries can be directed to the corresponding author. The nucleotide sequences of *ITS* (PV545913: https://www.ncbi.nlm.nih.gov/nuccore/PV545913, accessed on 21 April 2025), *ACT* (PV553737: https://www.ncbi.nlm.nih.gov/nuccore/PV553737, accessed on 21 April 2025), and *CHS* (PV553736: https://www.ncbi.nlm.nih.gov/nuccore/PV553736, accessed on 21 April 2025) generated in this study were deposited in GenBank database.

## Supplementary Materials

The following supporting information can be downloaded at https://www.mdpi.com/article/10.3390/jof11090633/s1, Figure S1: Phylogenetic analysis of *C. kahawae* based on the combined sequence dataset of *ITS* by Maximum Likelihood method. Figure S2: Phylogenetic analysis of *C. kahawae* based on the combined sequence dataset of *ACT* by Maximum Likelihood method. Figure S3: Phylogenetic analysis of *C. kahawae* based on the combined sequence dataset of *CHS* by Maximum Likelihood method. Table S1: PCR primers used in this study; Table S2: Strains used in this study; Table S3: Sequence information and comparative analysis of *C. kahawae* and *C. liriopes*.
