# Supplementary material for: First Report of Colletotrichum kahawae Causing Anthracnose on Buckwheat (Fagopyrum tataricum) in China and Biological Characterization of the Pathogen
Source: J Fungi (Basel). 2025 Aug 29;11(9):633. doi: 10.3390/jof11090633 (PMC12470724; doi:10.3390/jof11090633)
Supplement: Supplementary file 1 [file jof-11-00633-s001.zip › jof-3730656-supplementary.pdf]

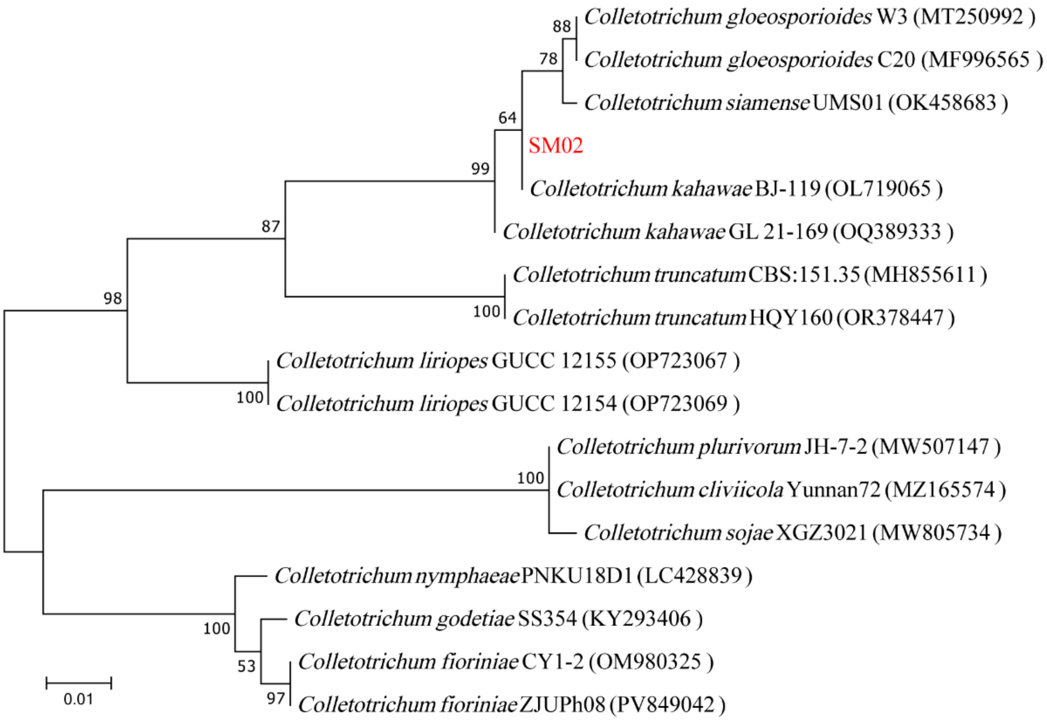

Supplementary Figure S1. Phylogenetic analysis of *C. kahawae* based on the combined sequence dataset of *ITS* by Maximum Likelihood method. The phylogenetic tree was generated using MEGA 7.0 software with 1000 bootstrap replicates. The red letter is the strain determined in this study.

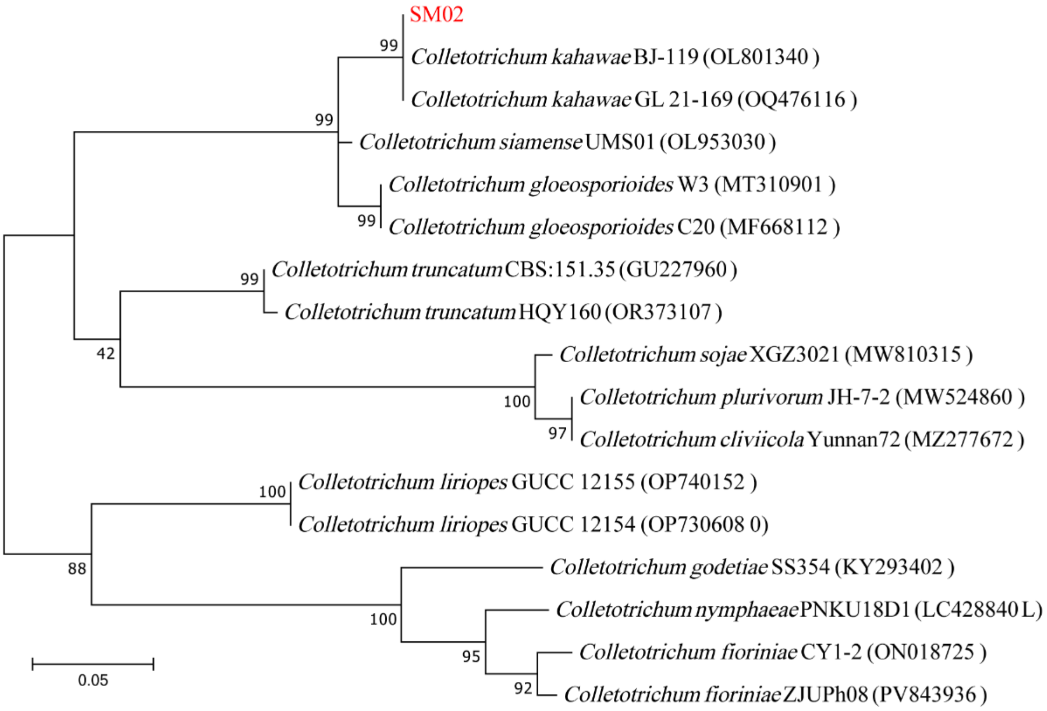

Supplementary Figure S2. Phylogenetic analysis of *C. kahawae* based on the combined sequence dataset of *ACT* by Maximum Likelihood method. The phylogenetic tree was

generated using MEGA 7.0 software with 1000 bootstrap replicates. The red letter is the strain determined in this study.

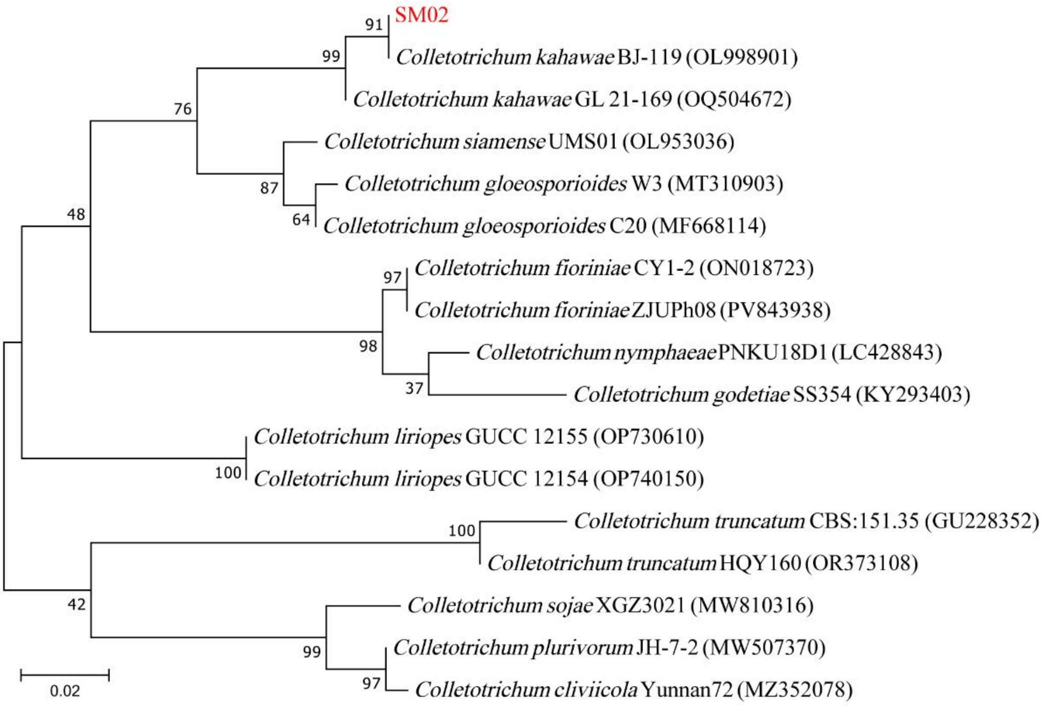

Supplementary Figure S3. Phylogenetic analysis of *C. kahawae* based on the combined sequence dataset of *CHS* by Maximum Likelihood method. The phylogenetic tree was generated using MEGA 7.0 software with 1000 bootstrap replicates. The red letter is the strain determined in this study.

Supplementary Table S1. Primers for PCR used in this study.

**Genes**	**Primer**	**Primer Sequence (5′→3′)**
ITS	ITS1	TCCGTAGGTGAACCTGCGG
	ITS4	TCCTCCGCTTATTGATATGC
ACT	ACT-512F	ATGTGCAAGGCCGGTTTCGC
	ACT-783R	TACGAGTCCTTCTGGCCCAT
CHS	CHS-79F	TGGGGCAAGGATGCTTGGAAGAAG
	CHS-345R	TGGAAGAACCATCTGTGAGAGTTG

Supplementary Table S2. Strains used in this study.

Original Name	Culture Accession Number(s)	GenBank accession		
		ITS	***ACT***	***CHS-1***
Colletotrichum kahawae	BJ-119	OL719065	OL801340	OL998901
Colletotrichum kahawae	GL 21-169	OQ389333	OQ476116	OQ504672
Colletotrichum siamense	UMS01	OK458683	OL953030	OL953036
Colletotrichum gloeosporioides	W3	MT250992	MT310901	MT310903
Colletotrichum gloeosporioides	C20	MF996565	MF668112	MF668114
Colletotrichum truncatum	CBS:151.35	MH855611	GU227960	GU228352
Colletotrichum truncatum	HQY160	OR378447	OR373107	OR373108
Colletotrichum plurivorum	JH-7-2	MW507147	MW524860	MW507370
Colletotrichumcliviicola	Yunnan72	MZ165574	MZ277672	MZ352078
Colletotrichum sojae	XGZ3021	MW805734	MW810315	MW810316
Colletotrichum godetiae	SS354	KY293406	KY293402	KY293403
Colletotrichum nymphaeae	PNKU18D1	LC428839	LC428840	LC428843
Colletotrichum fioriniae	CY1-2	OM980325	ON018725	ON018723
Colletotrichum fioriniae	ZJUPh08	PV849042	PV843936	PV843938
Colletotrichum liriopes	GUCC 12155	OP723067	OP740152	OP730610
Colletotrichum liriopes	GUCC 12154	OP723069	OP730608	OP740150

Supplementary Table S3. Sequence information and comparative analysis of *C. kahawae* and *C. liriopes*.

Genes	Alignment length	Best match accession	Identity (%)	Substitution model	Node support values
ACT	254	KY995503	80.43%	Tamura-Nei	100
CHS	295	KY995452	90.17%	Tamura-Nei	100
ITS	615	MZ314513	93.07%	Tamura-Nei	100

Note: The parameters of substitution model and node support values from maximum likelihood phylogenetic analysis.
